# Sporadic Burkitt Lymphoma of the Thoracic and Lumbar Spinal Canal in an Adult: Oncogenicity and a Literature Review

**DOI:** 10.7759/cureus.26860

**Published:** 2022-07-14

**Authors:** Bahadar S Srichawla

**Affiliations:** 1 Department of Neurology, UMass Chan Medical School, Worcester, USA

**Keywords:** epidural, spinal canal, spinal cord, ivac, codox, sporadic burkitt lymphoma, burkitt lymphoma, radiology, oncology, neurology

## Abstract

Burkitt lymphoma (BL) is an aggressive form of lymphoma that occurs due to translocation of the *c-myc *proto-oncogene on chromosome 8. BL is characterized by three distinct groups: African/endemic variant, immunosuppressive variant, or sporadic variant. Most cases of the sporadic variant occur in patients less than 40 years of age with a median age of 30 at diagnosis and are primarily seen in Caucasians. An immunocompetent 69-year-old male presented with subacute onset weakness in the lower extremities. Magnetic resonance imaging (MRI) of the lumbar spine revealed a mass in the right paraspinal musculature with epidural extension, neural foraminal narrowing, and severe spinal canal stenosis in L2-L5. MRI of the thoracic spine revealed significant T5-T6 cord compression due to metastatic masses. Further diagnostic imaging revealed diffuse lymphadenopathy within the mediastinum and abdomen. Subsequently, the patient underwent a core needle biopsy of the left axillary lymph node, which revealed cluster of differentiation 20 and 10 (CD20 and CD10), *c-myc*, and B-cell lymphoma 6 (Bcl-6) positive lymphoid cells. A diagnosis of BL was made. The patient was treated with oral steroids and received one round of radiation therapy. The patient opted to forgo any antitumor treatment and was discharged to hospice. Primary lymphomas of the central nervous system (CNS) account for <5% of all CNS tumors. Approximately 5-10% of CNS lymphomas are recorded as BL, with the majority classified as high-grade B-cell lymphomas. Paraspinal involvement with BL is rare and not commonly seen in the sporadic variant.

## Introduction

Burkitt lymphoma (BL) occurs due to translocation t(8;14) involving the *c-myc *proto-oncogene on chromosome 8 and heavy-chain immunoglobulin (Ig) locus on chromosome 14 in 75% of cases [1]. BL is seen in three distinct forms. The African/endemic variant is commonly seen in Africa and South America and is often secondary to the Epstein-Barr virus (EBV). The endemic variant typically presents with a mass in the maxillary and mandibular bones. The immunosuppressive variant is often seen secondary to infection with the human immunodeficiency virus (HIV). The sporadic variant is commonly seen in Caucasians and has a median age of 30 years for onset. The sporadic variant typically presents with a mass in the abdomen or pelvis [1].

Primary central nervous system lymphoma (PCNSL) accounts for fewer than 5% of all central nervous system (CNS) tumors. Only approximately 5-10% are recorded as BL. PCNSL within the brain parenchyma leads to focal neurologic deficits in approximately 70% of patients. Symptoms may include nausea, vomiting, headache, and other signs of increased intracranial pressure (ICP). Lymphoma that affects the spinal cord leads to variable focal neurologic deficits depending on the anatomy of the tumor; sensory symptoms are more commonly seen. However, muscle weakness, bowel, and bladder dysfunction are not uncommon [2]. Reported here is a case of primary sporadic BL affecting the lumbar spinal canal in an adult. A literature review of similar cases of BL involving the spinal cord is included. Further points on treatment and outcomes are discussed.

## Case presentation

A 69-year-old male presented to the emergency department with a two-week history of progressively worsening lower extremity weakness. The patient stated that he previously could walk without assistance. However, now he required a walker to ambulate. He denied any stool or urinary incontinence. The patient denied using any previous or current recreational drugs. Vital signs on admission included a temperature of 35.7°C, heart rate of 84 beats per minute, blood pressure of 124/87 mmHg, and oxygen saturation of 96% on room air via pulse oximetry. A comprehensive neurologic examination was completed revealing 3/5 muscle strength of the bilateral lower extremities, 3+ hyperreflexia of the bilateral patellar tendons, downward Babinski reflex, no ankle clonus, and normal sensation to both vibratory and pin-prick sensation. The patient had a normal rectal tone and negative Romberg test.

**Figure 1:**
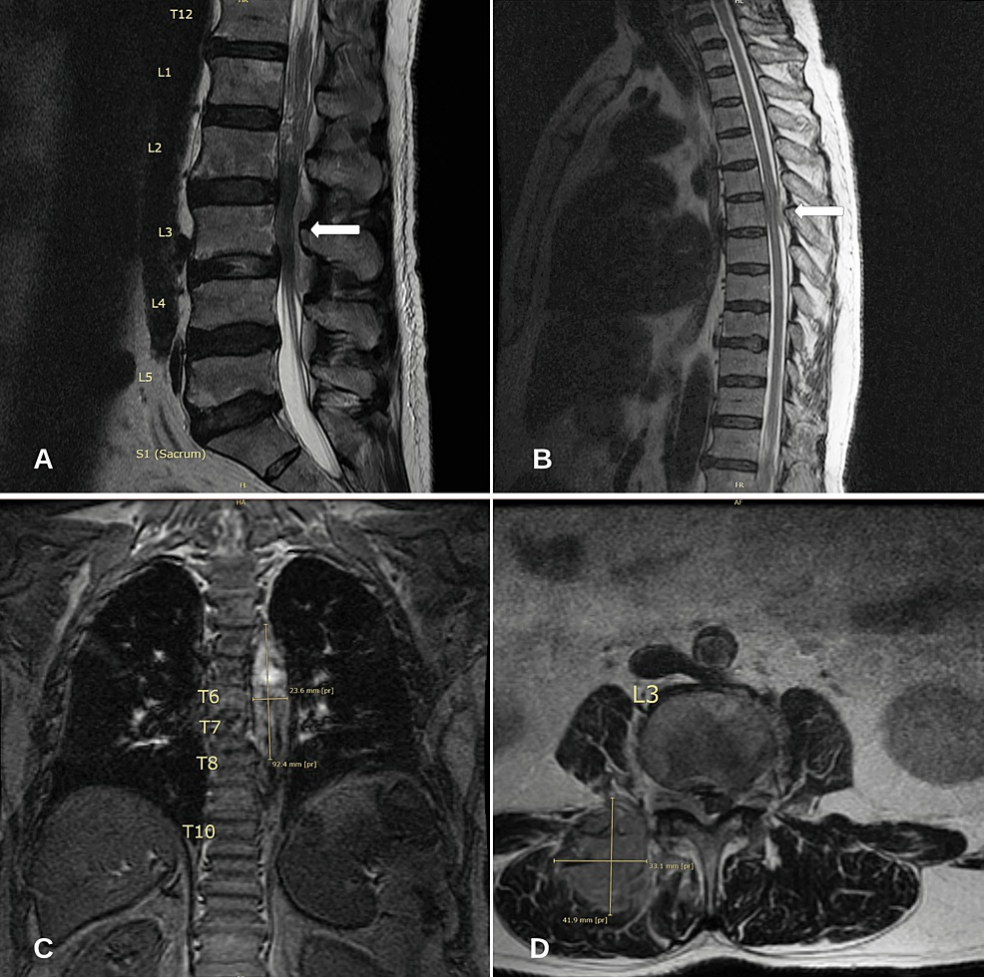
MRI of the thoracic and lumbar spine. (A) MRI T2 sagittal view of the lumbar spine revealing L2-L5 spinal canal stenosis. (B) MRI T2 sagittal view of the thoracic spine revealing significant cord compression at T5-T6 due to ventral and dorsal epidural masses. (C) MRI T2 coronal view of the thoracic spine revealing heterogeneous masses measuring 9.2 × 2.3 cm likely representing metastatic disease. (D) MRI T2 axial view of the lumbar spine revealing a mass measuring approximately 3.1 × 4.1 cm in the right paraspinal musculature with direct epidural extension and neural foraminal narrowing. MRI: magnetic resonance imaging

**Figure 2:**
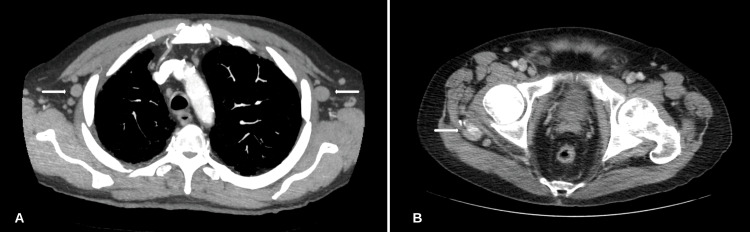
CT scan of the chest, abdomen, and pelvis. (A) Bilateral axillary lymphadenopathy. (B) A 1.0 cm rounded soft tissue nodule along the fascial plane between the right gluteus medius and maximus muscles is significant for metastatic disease. CT: computerized tomography

A comprehensive metabolic profile (CMP) and complete blood count (CBC) revealed no abnormalities. Lactate dehydrogenase (LDH) was recorded at 1,283 U/L (81-234), phosphorus of 6.2 mg/dL (2.5-4.5), and uric acid (UA) at 7.6 mg/dL (2.0-8.0). A urine and serum toxicology screening test was negative. Magnetic resonance imaging (MRI) of the lumbar spine with and without contrast was obtained and revealed a right paraspinal tissue mass with direct neural foraminal involvement, epidural extension, and severe spinal canal stenosis at L2-L5. MRI of the thoracic spine revealed compression of the cord at T5-T6 due to ventral and dorsal epidural masses (Figure 1). Given the significant concern for a neoplastic process, a computerized tomography (CT) scan of the chest was completed revealing bilateral axillary lymphadenopathy. A CT scan of the abdomen and pelvis was completed revealing multi-station lymphadenopathy and scattered sclerotic osseous metastasis (Figure 2).

Further infectious workup with blood and urine cultures and severe acute respiratory syndrome coronavirus 2 (SARS-CoV-2) RNA via polymerase chain reaction (PCR) test was negative. HIV, hepatitis B virus (HBV), hepatitis C (HCV), and cytomegalovirus (CMV) antibody tests were negative. EBV capsid antigen IgG antibody was >750.00 (>21.99 positive) in serum. EBV capsid antigen IgM antibody was <36.00 (<36.00 negative).

**Figure 3:**
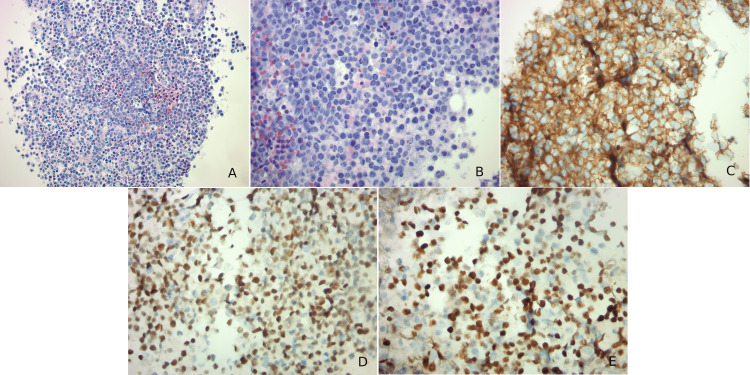
Histology and immunohistochemistry of the left axillary lymph node biopsy. (A) Medium-sized lymphoid cells with many mitotic figures. There are numerous tangible body macrophages admixed within the lymphoid cells. (B) High-power image of the previous slide. (C) Immunohistochemical stain is positive for cluster of differentiation 10 in blue cells. (D) Immunohistochemical stain is positive for B-cell lymphoma 6 in blue cells. (E) Immunohistochemical stain is positive for *c-myc* in blue cells.

The patient underwent a core-needle biopsy of the left axillary lymph node which revealed cluster of differentiation 20 and 10 (CD20 and CD10), *c-myc*, and B-cell lymphoma 6 (Bcl-6) positive lymphoid cells on the immunohistochemical stain. Microscopic examination revealed lymphoid cells with many mitotic figures and numerous tangible body macrophages (Figure 3). The Ki-67 proliferation index was greater than 90%. Fluorescence *in situ* hybridization (FISH) studies revealed a MYC/immunoglobulin heavy (IGH) rearrangement while negative for the B-cell lymphoma 2 (Bcl-2) rearrangement. A diagnosis of BL was made.

The patient was started on oral dexamethasone 4 mg every six hours with concern for spinal cord compression. He received one round of palliative radiation therapy with 3,000 centigray (cGy) in 3,000 cGy fractions. The patient was deemed a nonsurgical candidate due to the risk of worsening neurologic deficits and wide-spread metastasis. A palliative care discussion was held, and the patient opted to forgo any antitumor treatment and was discharged to hospice. Neurological examination on the day of discharge included 4/5 muscle strength of the bilateral lower extremity and 3+ hyperreflexia of the patellar tendon.

## Discussion

**Figure 4:**
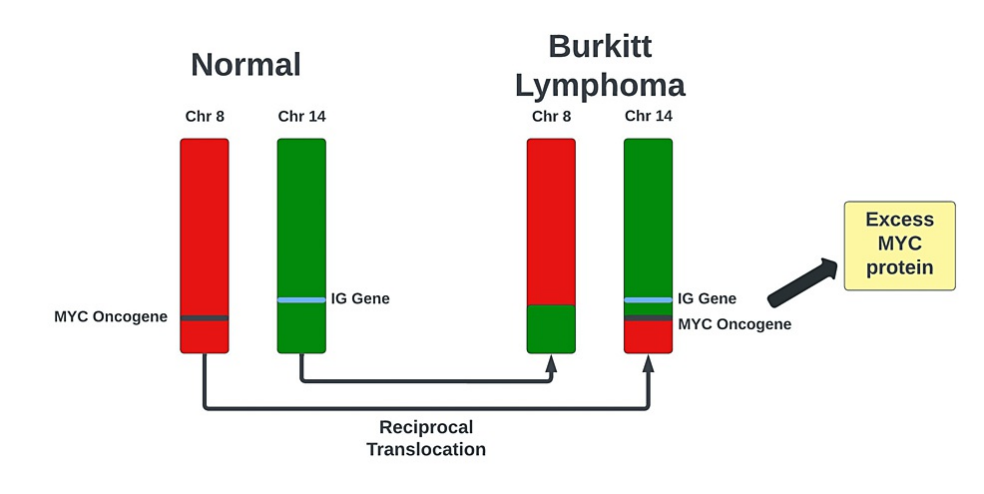
Reciprocal translocation t(8;14)(q24;q32) leading to the activation of the c-myc protoncogene. MYC: Myc Proto-oncogene. IG: immunoglobulin

BL is a cancer of B lymphocytes and is named after Denis Parsons Burkitt, a surgeon who first described the disease in 1958. It is classified into three distinct variants: endemic (African subtype), sporadic (non-African subtype), or immunosuppressed (acquired immunodeficiency syndrome-associated) [2]. Described here is a case of sporadic (non-African) BL in a 69-year-old male that presented as a lumbar paraspinal mass invading the epidural space and spinal canal. The patient was found to have metastatic disease with cord compression of the thoracic spine and diffuse lymphadenopathy. All variants of BL are associated with chromosomal translocation, with more than 85% of cases of BL attributed to t(8;14)(q24;q32) [3]. This reciprocal translocation involving the *c-myc* and IGH locus gene leads to the unregulated activation of the *c-myc* proto-oncogene causing excess protein production and increased cellular proliferation (Figure 4). Other genetic variants less commonly seen in BL are the t(2;8)(p12;q24) and t(8;22)(q24;q11) chromosomal translocations. Our patient was confirmed to have the t(8;14) chromosomal translocation as shown via FISH.

**Figure 5:**
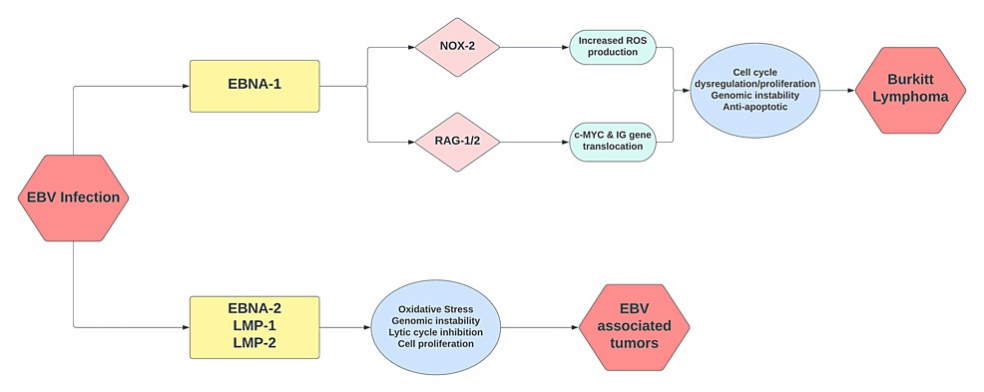
Oncogenic mechanisms associated with the EBV. EBV: Epstein-Barr virus; EBNA-1: Epstein-Barr virus nuclear antigen 1; EBNA-2: Epstein-Barr virus nuclear antigen 2; LMP-1: latent membrane protein 1; LMP-2: latent membrane protein 2; NOX-2: reduced nicotinamide adenine dinucleotide phosphate oxidase 2; RAG-1/2: recombination activating gene 1/2; IG: immunoglobulin; c-MYC: cellular-Myc proto-oncogene

The EBV or human herpesvirus 4 (HHV4) is the cause of infectious mononucleosis. After primary infection with the virus, it remains latent within B lymphocytes and can encourage neoplastic proliferation of these cells [4]. EBV has been detected in approximately 30% of cases involving the sporadic/non-African variant of BL and 25-40% of BL due to immunodeficiency [5]. Almost 98% of endemic/African BL is associated with EBV. In our case, the EBV capsid antigen IgG antibody was >750.00 (>21.99 positive) in serum. EBV capsid antigen IgM antibody was <36.00 (<36.00 negative). This is indicative of a prior infection with EBV which may have played a role in the neoplastic proliferation of lymphoid cells via t(8;14)(q24;q32) confirmed by FISH (Figure 5).

Most cases of sporadic BL present with abdominal masses and lymphadenopathy with the typical presentation being abdominal distension. In our case, the patient was found to have diffuse lymphadenopathy that included the abdomen. However, the large paraspinal lumbar mass was the location of the neoplasm with heterogeneous masses in the thoracic spine evidence of metastatic disease. The chief complaint of lower extremity weakness and hyperreflexia is attributed to neural foraminal involvement in the lumbar spine and possibly cord compression in the thoracic spine (T5-T6). Other causes of spasticity from spinal cord lesions include subacute combined degeneration from vitamin B_12_ deficiency [6]. The endemic form of BL is known to have favorable outcomes, being highly responsive to chemotherapy. The sporadic and immunocompromised variants of BL are not as chemo-responsive as the endemic/African variant [4]. Furthermore, our patient had signs of a poor prognosis based on metastatic disease evident in diffuse lymphadenopathy, poor functional status, and elevated LDH. Other poor prognostic factors seen included prominent CNS involvement [4,7]. Tumor lysis syndrome (TLS) can occur spontaneously and is characterized by hyperkalemia, hyperuricemia, hyperphosphatemia, and hypocalcemia [8]. In our patient, hyperuricemia and hyperphosphatemia were present. Our patient had chosen to forego antitumor treatment and proceeded with palliative measures only.

Treatment options for BL include intensive chemotherapy regimens. Prominent treatment options include a cocktail regimen that includes cyclophosphamide, doxorubicin, vincristine, methotrexate, ifosfamide, cytarabine, and etoposide commonly referred to as CODOX-M/IVAC. This regimen is often supplemented with high-dose cytarabine and intrathecal methotrexate (MTX) for CNS prophylaxis [9]. Disease progression with CNS involvement is not uncommon, and an anticipated 30-50% of patients develop CNS lesions with leptomeningeal disease being more common than parenchymal disease [10]. Other treatment regimens used for BL include rituximab, etoposide phosphate, prednisone, vincristine sulfate (Oncovin), cyclophosphamide, and doxorubicin hydrochloride (hydroxydaunorubicin) also known as R-EPOCH.

Literature review

A literature review was conducted using PubMed/PubMed Central/MEDLINE database. The following search string was utilized: (“Burkitt lymphoma”) AND (“spinal cord” OR “spinal canal”). The analysis only included adult cases of BL with primary spinal involvement. Five cases were identified and are included in Table 1.

**Table 1:** Summary of reported cases of adult Burkitt lymphoma with spinal involvement. CODOX-M/IVAC: cyclophosphamide, vincristine, doxorubicin, high-dose methotrexate/ifosfamide, etoposide, high-dose cytarabine; MTX: methotrexate; R-CHOP: rituximab, cyclophosphamide, hydroxydaunomycin, oncovin, prednisolone; R-EPOCH: rituximab, etoposide, prednisolone, oncovin, cyclophosphamide, hydroxydaunorubicin

Author(s)	Age	Gender	Presenting symptoms	Type	Location	Intervention	Outcomes
Bansal et al. [11]	47	M	Neck pain, quadraperesis	Sporadic	Cervical	CODOX-M/IVAC	Improved symptoms
Kim et al. [12]	69	F	Back pain	Sporadic	Lumbar	Hemilaminectomy, rejected chemotherapy	Non-improved
Wilkening et al. [13]	43	F	Sciatica	Sporadic	Lumbar	Intrathecal MTX and systemic cyclophosphamide, vincristine, methotrexate, ifosfamide, adriamycin, and dexamethasone	Improved symptoms
Malani et al. [14]	53	M	Back pain, right lower extremity weakness	Sporadic	Thoracic	Laminectomy and CODOX-M/IVAC	Resolution of radiographic findings, improved symptoms
Neel et al. [15]	73	F	Back pain	Sporadic	Lumbar	Laminectomy and radiation therapy. Intrathecal MTX and cytarabine. Systemic R-CHOP and R-EPOCH	Limited improvement. Relapse in five months

Bansal et al. (2022) reported a case of a 47-year-old male with progressive neck pain, quadriparesis, and bladder incontinence for a period of five months. MRI findings were significant for extramedullary lesions in the vertebral section C1-C2. Surgical excision of the site and immunohistochemical staining revealed CD45, CD20, and Bcl-6 positive lymphoid cells but negative for Bcl-2 and EBV. The patient was diagnosed with BL and treated with CODOX-M/IVAC with variable outcomes [11].

Kim et al. (2015) reported a case of a 69-year-old woman with a one-month history of back pain prior to presentation. MRI revealed a dumbbell-shaped epidural mass that extends into the L2-L3 vertebral section. Tumor resection and immunohistochemical staining revealed CD10, CD20, Bcl-6, and EBV-positive cells while negative for Bcl-2. The patient underwent hemilaminectomy for tumor removal but rejected further chemotherapy [12].

Wilkening et al. (1997) reported a case of a 43-year-old woman with a four-day history of lower back pain and sciatica. The initial MRI of the spine revealed no abnormalities; however, a repeat MRI of the lumbar spine six weeks later revealed an epidural mass at the L2-L3 vertebral section. Histological examination revealed CD20-positive cells. The patient was treated with local radiation and polychemotherapy with intrathecal MTX and systemic cyclophosphamide, vincristine, methotrexate, ifosfamide, adriamycin, and dexamethasone. The patient was reported to have a slow recovery [13].

Malani et al. (2000) reported a case of a 53-year-old male with scapular back pain, right lower extremity weakness, and numbness for one month. An MRI revealed an epidural mass in the T4-T10 section of the thoracic spine. Immunohistochemical staining of the tumor section revealed CD10, CD20, CD43, and CD45 positive cells while negative for CD5, CD23, and Bcl-2. A high proliferation rate (Ki-67> 98%) was present (Ki-67 >98%) and the flow cytometry results were consistent with BL. The patient underwent four cycles of chemotherapy with CODOX-M/IVAC. Complications with treatment included MTX-mediated renal failure and neutropenic fever. Repeat MRI showed resolution of the epidural mass and favorable functional outcomes [14].

Neel et al. (2020) reported a case of a 73-year-old female who presented with lower back pain for one week and bilateral chin numbness for two weeks. MRI revealed spinal canal involvement in the L2-L3 segments of the lumbar spine with compression of the cauda equina. The patient underwent laminectomy, five rounds of radiation therapy, and one round of rituximab, cyclophosphamide, hydroxydaunomycin, oncovin, prednisolone (R-CHOP), intrathecal cytarabine, and MTX. The patient also received one round of rituximab, etoposide, prednisolone, oncovin, cyclophosphamide, and hydroxydaunorubicin (R-EPOCH). The patient represented five months later with relapse of lymphoma and expired on comfort measures [15].

## Conclusions

A case of sporadic BL is reported in a 69-year-old immunocompetent male with a primary presentation of lower extremity weakness. He was found to have a right paraspinal mass invading the lumbar spinal canal with epidural and neuro-foraminal narrowing. In addition, he had metastatic disease with diffuse lymphadenopathy and thoracic cord compression. The diagnosis was confirmed via histology (high mitotic index), immunohistochemical staining (CD 20, CD10, Bcl-6 positive, Bcl-2 negative), and FISH showing MYC and IGH rearrangement. Our patient opted to forego antitumor treatment and was discharged to hospice. A literature review of adult cases of sporadic BL with primary spinal cord involvement is included. Treatment options including CODOX-M/IVAC, intrathecal MTX, and cytarabine are discussed. BL remains a rare PCNSL.
